# Developing an innovation and enterprise framework for translating UK-driven global health research into commercially viable interventions: the FLIGHT study protocol

**DOI:** 10.1371/journal.pone.0323168

**Published:** 2025-05-13

**Authors:** Ezekiel Boro, Charles McLoughlin, Tom Vaughan, Carolina Velasco, Lisa Tilokani, Katherine Prescott, Gail Davey, Simon J. Waddell, Alexandra Anderson, Justin Pulford, Chris Peters, Becky Jones-Phillips

**Affiliations:** 1 Enterprise and Innovation Department, Liverpool School of Tropical Medicine, Liverpool, United Kingdom; 2 Research Strategy and Knowledge Exchange Office, Liverpool School of Tropical Medicine, Liverpool, United Kingdom; 3 Strategic Research Office, London School of Hygiene and Tropical Medicine, London, United Kingdom; 4 Department of Global Health and Infection, Brighton and Sussex Medical School, University of Sussex, Brighton, United Kingdom; 5 Department of International Public Health, Liverpool School of Tropical Medicine, Liverpool, United Kingdom; PLOS: Public Library of Science, UNITED KINGDOM OF GREAT BRITAIN AND NORTHERN IRELAND

## Abstract

The UK Higher Education Institution (HEI) sector faces significant challenges in translating early-stage research into commercially viable public health products, particularly in infectious diseases. This gap, often termed the “valley of death,” is exacerbated by a fragmented ecosystem and unclear pathways to implementation. The FLIGHT (Framework for Leveraging Innovation in Global Health Technologies) project aims to address these challenges by enhancing the commercialisation of global health research from UK HEIs to drive economic and societal impact. Led by Liverpool School of Tropical Medicine, in partnership with London School of Hygiene and Tropical Medicine, Brighton and Sussex Medical School, and other associated partners, FLIGHT seeks to develop a best practice Innovation and Enterprise Framework (IEF) to support R&D commercialisation. This project will use a mixed-methods approach to assess existing public health R&D assets and the commercialisation knowledge and skills of HEI professionals and Early Career Researchers (ECRs) across partner institutions. Data collection will include literature reviews, surveys, qualitative interviews, and focus group discussions exploring staff and ECR’s perceptions and experiences with commercialisation, to identify barriers and opportunities for improvement. Ethical considerations and participant confidentiality are central to the study design. The FLIGHT project aims to bridge critical gaps in the innovation pipeline, fostering a robust ecosystem for translating HEI research into deployable health interventions. This initiative will contribute to strengthening the UK’s global health R&D commercialisation capabilities and accelerating the development and deployment of essential public health interventions. Findings will inform policy recommendations and best practice for HEI commercialisation strategies, with implications for both UK and low-and middle-income country (LMIC) contexts.

## Introduction

The UK’s research and Higher Education Institution (HEI) sector is recognized as a global leader in scientific knowledge generation [1]. However, compared to other Organisation for Economic Co-operation and Development (OECD) countries, it is losing its competitive edge in translating ideation and early-stage research and development (R&D) into commercially viable products that can drive significant economic and societal impact [1]. For example, developing new products for public health in the infectious disease field is challenging and under-invested in the UK [2]. There is also unmet need in the later stages of product development and market access of global public health interventions, where improving regulatory navigation and building compelling health economic models to engage NHS and Global Healthcare payors is of critical importance [3].

In the past 20 years, the world has faced numerous public health emergencies of international concern arising from infectious diseases [4]. The Ebola epidemic and COVID-19 pandemic for instance have highlighted the importance of having agile frameworks for research translation that consider not only clinical effectiveness, but also cost effectiveness and the regulatory environment, as well as the feasibility and acceptability of adoption of interventions into health systems [5]. The UK HEI system plays a key role in driving and sustaining translational R&D activity; however, UK infectious disease research assets of global importance may be overlooked by HEI Technology Transfer Office (TTO) systems of commercialisation, primarily due to the lack of specific domain expertise and a responsive, rather than proactive, model for TTOs. Successful conversion of R&D HEI assets will not only enable robust mitigation strategies for future epidemics and pandemics globally, but will contribute to the economic growth of the Science, Technology, and Innovation (STI) sector in the UK [6].

The **Framework for Leveraging Innovation in Global Health Technologies (FLIGHT)** consortium comprises three UK HEI delivery partners and ten associated partners from the health and life sciences sector globally. The UK HEI partners are – Liverpool School of Tropical Medicine (LSTM), London School of Hygiene and Tropical Medicine (LSHTM) and Brighton and Sussex Medical School (BSMS). The FLIGHT project aims to explore and improve how the commercialisation of global health-facing research can be better supported across the HEI partners, and to develop a best practice framework for other universities with specialist focused research portfolios orientated around social impact. The consortium partners have complementary expertise and internationally recognised track records in infectious diseases and global health research impact, and this will be leveraged to develop a model of best practice for UK-wide and LMIC deployment.

**Fig 1:**
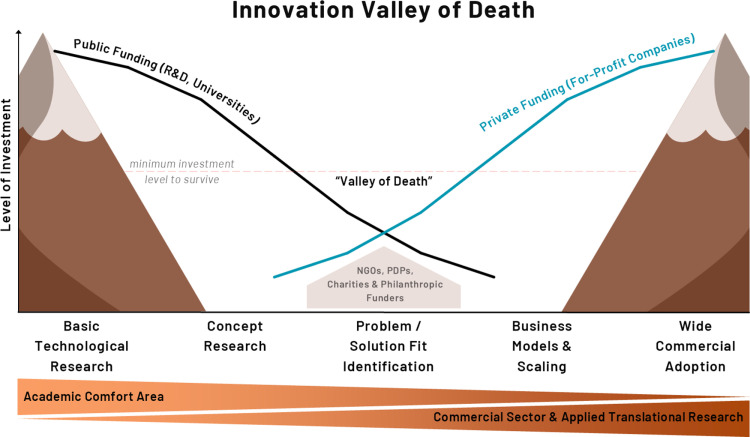
Innovation Valley of Death. NGOs = Non-Governmental Organisations, PDPs = Product Development Partnerships.

A key focus of the project is enhancing the support provided for commercialisation of research outputs and addressing roadblocks present at the typical early to mid-stages (often referred to as the ‘valley of death’) in the innovation pipeline, where the current ecosystem is fragmented and pathways to adoption/implementation are unclear (Fig 1) [7,8].

The FLIGHT project will provide insights and tools to address key valley of death and late-stage innovation challenges, primarily by: addressing identified gaps early in the translational journey; leveraging global expertise to develop solutions to the challenges presented; de-risking onward investment; and ultimately expediting development of global public health interventions. A major output will be an Innovation and Enterprise Framework (IEF) that will act as a blueprint for commercial acceleration, market access and to ultimately deliver a step-change to overcome challenges within the current ecosystem.

### Overarching goal(s) of the FLIGHT project

**Fig 2:**
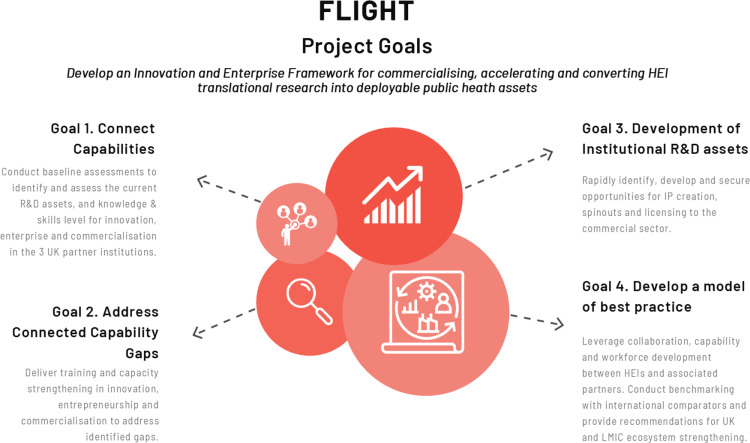
FLIGHT Project Goals.

***To develop an Innovation and Enterprise Framework for commercialising, accelerating and converting HEI translational research into deployable public health assets*** by seeking to:

- Goal 1. *Connect capabilities*: Conduct baseline assessments to identify and assess the current R&D assets, and knowledge & skills level for innovation, enterprise and commercialisation in the 3 UK partner institutions (Fig 2).
- Goal 2. *Address connected capability gaps*: Deliver training and capacity strengthening in innovation, entrepreneurship and commercialisation to address identified gaps (Fig 2).
- Goal 3. *Development of institutional R&D assets*: Rapidly identify, develop and secure opportunities for IP creation, spinouts and licensing to the commercial sector (Fig 2).
- Goal 4. *Develop a model of best practice*: by leveraging collaboration, capability and workforce development between HEIs and associated partners. Conduct benchmarking with international comparators and provide recommendations for UK and LMIC ecosystem strengthening (Fig 2).

## Materials and methods

The methods described below focus on the baseline assessments to be conducted as part of goal 1 described above. The insights and data from the baseline assessments will be used to develop and implement a range of interventions, including training programs and policy and process enhancements at partner HEIs (goal 2). A successful implementation of these interventions will improve the identification and commercialisation of R&D assets for licensing and spin-out opportunities across our institutions (goal 3) and position our FLIGHT Innovation and Enterprise framework as a best practice model for both UK and global institutions (goal 4).

### Study design

We will use a mixed-methods approach including scientific and grey literature review, surveys, qualitative interviews and focus groups with key informants and quantitative data analysis.

### Study objectives

In the three partner institutions we will:

1. Assess existing public health and infectious disease R&D institutional assets.
2. Assess the knowledge and skills level of academic and non-academic professional staff on innovation, entrepreneurship and commercialisation.

To achieve study objective 1, we will conduct qualitative scientific and grey literature review and quantitative analysis of secondary data sources such as Scopus, SciVal, UK, EU and WIPO (World Intellectual Property Organization) Patent Office database, Pitch Book, PubMed, UK government and partner HEI (network) websites and internal reports, policies and other documents.

To achieve study objective 2, we will conduct surveys, qualitative interviews and focus groups with key informants across all partner HEIs, to be led by the research teams at each partner institution.

#### Survey.

We will conduct an electronic survey (e-survey) across partner institutions. Participants will be recruited via email invitations sent through institutional mailing lists. We anticipate a minimum of ~ 50 respondents at each HEI. The email will include a brief description of the project and a link to the online survey. Reminders will be sent two weeks after the initial invitation to encourage participation.

All institutional staff who can communicate in the English language and are currently employed part-time or full-time with a temporary or permanent contract as teaching, research, or professional staff at each partner institution will be eligible to participate. A detailed participant information section explaining the study’s purpose, procedures, risks, and benefits will be included in the e-survey. An informed consent section must also be completed before accessing the survey. The survey will be administered electronically using a Microsoft Form and will include Likert-scale questions assessing confidence levels across various pre-defined themes and some open-ended questions to allow respondents to elaborate on some of their responses. The survey will be open for four weeks and responses will be collected anonymously. Please see S1 File for a copy of the survey questionnaire.

#### Key informant interviews.

We will conduct semi-structured interviews with academic (research and teaching) and non-academic professional staff at LSTM, LSHTM, and BSMS. Interviewees will include teaching staff (lecturer and other cadres above), principal investigators (PIs) and/or research group leaders (RGL) and non-academic professional staff with, e.g., research management services, operations, finance and contracting responsibilities.

Interviews will be based on an interview guide developed to gather insights across 5 themes: ‘Understanding and Experience’, ‘Reward, Recognition and Incentives’, ‘Policy and Processes’, ‘Sustainability of Commercialisation Initiatives’, and ‘Knowledge of Enterprise and Innovation’. Participants will be recruited via personal institutional email invitations and internal communications and marketing campaigns and channels at the three HEIs. A detailed participant information sheet will be provided to potential participants before interviews, explaining the study’s purpose, procedures, risks, and benefits. Participants must provide written informed consent before participating in interviews. Interviews will be done in-person or via online audio/video conferencing software (Microsoft Teams) at a pre-agreed time. Interviews will be conducted in the English language, will take ~60 minutes and will be audio or video recorded (both in person and virtually using Microsoft Teams). Interviews will be recorded in full and transcribed.

Participation is voluntary, and participants can withdraw at any time without penalty. Withdrawal from the key informant interviews will be maintained by adhering to the following methods:

1 *Participant right to withdraw*:
  Participants will be reminded that they can withdraw from the study at any time, without providing a reason. The withdrawal can be requested up until the data analysis phase. After analysis begins, full anonymisation will be in place, making it difficult to identify individual contributions.
2 *Procedure for data removal*:
  Upon withdrawal, any identifiable data associated with the participant will be removed from the study. This includes:

a Deleting any raw interview recordings (audio/video).
b Removing their responses from transcripts and analysis files.
c Ensuring that any portions of the data that have already been anonymised are excluded from the final analysis and not utilised.

If anonymisation has already been completed and the data cannot be linked to the participant anymore, removal will no longer be possible, but participants will be informed of this beforehand.

3 *Communication for withdrawal*:
  Dedicated contact emails at each study site (LSTM, LSHTM and BSMS) will be provided for participants to request withdrawal. A written confirmation will be sent once their data has been removed.

All participants may also be contacted following their interview to provide additional or confirmatory information.

Interview questions will relate to the themes outlined above, with no identifiable personal information required. We will ensure confidentiality and anonymity of the data provided and obtain written informed consent before conducting all interviews. Anonymity will be maintained post key informant interview by adhering to the following methods:

1. *De-identification of transcripts*: All names, institutions, and specific project details will be removed or replaced with generic terms during transcription. For example, if a participant refers to a specific innovation or partnership, this would be generalised as “a research project” or “a collaboration with external partners.”
2. *Code replacement*: In place of identifying phrases like “our work with the X vaccine,” or “the Y diagnostics trial,” generic terms such as “a public health intervention” or “a diagnostics study” will be used in transcripts.
3. *Moderation of overly specific responses*: If a response contains an excessive number of unique details that make it difficult to anonymise, the entire response will be excluded from the study. For instance, if a participant discusses a project that is easily identifiable due to its specificity within the institution, that segment of the interview will be redacted to protect anonymity.
4. *Generalised IP discussion*: In responses concerning intellectual property, specific examples of innovations or inventions will be avoided in favour of discussing broader principles or strategies employed in IP management.

By implementing these measures, the study will ensure that the anonymity of participants is maintained, while also allowing them full control over the use of their data

Please see S2 File, S3 File, S4 File respectively for copies of the interview guide, participant information sheet and consent form.

#### Sampling for key informant interviews.

Participants for the key informant interviews will be identified and selected using a convenience and purposive sampling technique based on the following criteria:

- ability to communicate in the English language
- currently employed part-time or full-time with a temporary or permanent contract as a lecturer (and other cadres above), principal investigator/research group leader (and other cadres above) or non-academic professional (manager and other cadres above) staff at each partner institution
- has worked at the institution for a minimum of 4 years

Exclusion criteria include:

- teaching staff below Lecturer level, research staff below PI/RGL level and non-academic professional staff below manager level
- teaching, research or non-academic professional staff with less than 4 years institutional working experience

Key informants who meet the above criteria may be further purposively selected if necessary to ensure gender equity and representation across the various categories of staff who are eligible. We hope to recruit ~30 participants across the teaching, research and/or professional staff in each institution, however, we may stop recruiting participants when we believe data saturation is achieved. We will determine data saturation by thematically coding and analysing these data iteratively during the data collection process to identify recurring themes and assessing whether new information is still emerging.

#### Focus groups.

Focus groups will also be conducted with post-doctoral and doctoral post graduate researchers at partner universities. Participants will engage in a focus group discussion alongside 6–10 others, sharing insights on their understanding and experiences of commercialisation processes as a route to impact. This input will help identify areas where additional support could be introduced or strengthened for staff interested in commercialising their research.

A detailed participant information sheet will be provided before the focus groups are scheduled, explaining the study’s purpose, procedures, risks, and benefits. Participants must provide informed consent before participating in focus groups. Participation is also voluntary, and participants can withdraw at any time. We will conduct a minimum of 2 focus groups, each with 6–10 participants at each institution. Each focus group will last ~1.5–2 hours and will be held in a convenient, private and safe setting in person or via a secure online platform (Microsoft Teams). Focus groups will be facilitated by a moderator who will guide the discussion using a semi-structured interview guide. Discussions will be audio or video recorded (both in person and virtually using Microsoft Teams) and transcribed. Please see S5 File for a copy of the focus group interview guide. A similar participant information sheet and consent form will be used for focus groups and interviews.

Focus group participants will be identified and selected using a convenience and purposive sampling technique based on the following criteria in Table 1:

Key informants who meet the above criteria may be further purposively selected to ensure gender equity and representation across the sample pool and categories of post-docs and PhD students who are eligible.

**Table 1:** Criteria for identifying and selecting focus group participants.

*Category*	*Inclusion criteria*	*Exclusion criteria*
Post-doctoral researchers	• ability to communicate in the English language • currently employed part-time or full-time with a temporary or permanent contract as a post-doctoral research/teaching staff at one of the partner institutions • has worked at the institution for a minimum of 2 years	• post-doctoral researcher with less than 2 years institutional working experience
Doctoral researchers (PhD students)	• ability to communicate in the English language • currently enrolled part-time or full-time as a doctoral student at one of the partner institutions • has studied at the institution for a minimum of 2 years	• doctoral student enrolled at institution for less than 2 years

### Study status and timeline

Participant recruitment and data collection for the study started on 2^nd^ December 2024 and will continue until 31^st^ March 2025. We hope to start and complete data analysis from 1^st^ April – 30^th^ June 2025. We expect study results to be finalised by August 2025.

### Data analysis and management

Quantitative data will be analysed using descriptive statistics (e.g., proportion, sum, mean, median, standard deviation) and inferential statistics (e.g., chi-square test, t-test) as appropriate. Some quantitative analysis may also be done for survey responses to close ended questions.

The research team will protect interview participants’ identity by anonymising contributions, assigning ID codes or pseudonyms when reporting individual quotes, and by not reporting specific job-titles. All qualitative data will be analysed thematically to identify common themes and patterns. NVivo or similar qualitative data analysis software will be used to assist with coding and analysis. We have also developed a rubric scoring matrix to analyse the qualitative data using a predefined set of descriptive criteria to score each question in the interview guide on a scale of 1 (low awareness) to 5 (high awareness), which will be summed to provide an overall awareness metric for each participant. Please see Table 2 below.

**Table 2:** Example rubric scoring matrix for the key informant interview question around academic readiness to engage with commercialisation, ranking answers as low awareness/understanding (1) to higher awareness/understanding (5).

Rubric Score	Description	Checkbox
**5**	The participant has clear suggestions for how to balance academic/professional outputs with engagement into commercialisation, believing a balance is crucial for career success.	☐
**4**	The participant has some ideas on how to prevent commercialisation activities from hindering academic/professional outputs but is unsure of their feasibility.	☐
**3**	The participant believes there is a moderate impact of commercialisation activities on academic/professional outputs but does not have strong ideas for change.	☐
**2**	The participant does not believe commercialisation activities significantly hinder academic/professional outputs.	☐
**1**	The participant believes that engaging in commercialisation activities impacts academic outputs and offers no suggestions on how to mitigate this from happening.	☐

#### Example interview question and associated rubric scoring.

“What would you like to happen so that as a Principal Investigator (PI) your academic outputs such as publishing are not hindered by engaging with research commercialisation activities?”

Interview and focus group recordings and transcripts will be stored securely on the OneDrive of a password-protected computer and in each institution’s FLIGHT team’s SharePoint folder and will only be accessible to the institution’s research team. Only anonymised versions of the transcripts will be shared with the wider FLIGHT study team across the three institutions. The recordings will be deleted immediately after transcription and other study materials (consent forms, transcripts, etc) will be stored securely for up to 10 years or for however long required before deletion in accordance with LSTM, LSHTM and BSMS’s respective Research Governance & Ethics Offices’ data storage procedures.

### Ethical considerations

This study will be conducted in accordance with the *Declaration of Helsinki (2008)* as well as in respect of the requirements set out in the applicable standard operating procedure of the study sponsor’s (LSTM) Research Ethics Committee (REC). We have obtained research ethics approval from LSTM’s REC for all study sites; in November 2024 for LSTM and BSMS and in January 2025 for LSHTM, protocol number 24–045.

Only data relevant to this study as outlined in this protocol will be collected by the research team. All information collected during the research project will remain confidential to the extent required and provided by law and policies at partner HEIs.

## Discussion

This study could have several limitations. Combining data from qualitative and quantitative sources can be difficult, especially if findings from the two methods appear to contradict each other or are challenging to reconcile. To address this potential challenge in integrating qualitative and quantitative data, we will employ a data triangulation approach, ensuring that findings are cross-validated and discrepancies explored in depth. Our mixed-method approach may also be subject to biases inherent in each method, such as selection bias in recruiting interview and focus group participants or response bias in surveys, which may affect the overall findings of the study. To minimize selection bias, we plan to use purposive sampling strategies that ensure diverse representation across participant demographics and key stakeholder groups.

Response bias will be mitigated by ensuring anonymity in survey responses and conducting interviews in neutral settings to encourage honest feedback. While the quantitative insights can be generalised in some instances, some of our qualitative findings will be generalisable and context-specific in some instances across the three study sites. Balancing generalised and context-specific insights based on the study results may be challenging. To enhance transferability of qualitative and quantitative insights, we will aim to provide rich contextual descriptions and detailed case studies in our final reporting to help readers understand the applicability of the findings in similar settings. Finally, managing ethical concerns, especially when handling sensitive qualitative data alongside broad quantitative data, can be complex and will require careful oversight. We are adhering to strict ethical guidelines, including obtaining written informed consent, ensuring confidentiality, and securely storing data in compliance with institutional requirements.

Findings from this study will be shared with the funder (Research England) and each institution’s administration through internal reports. Results will also be disseminated at national (UK HEIs, research & funder communities) and international levels (via scientific conferences and academic journals). All dissemination of data will follow the guidelines of the International Committee of Medical Journal Editors (ICMJE), and authorship will meet the ICMJE criteria for authorship.

Any study amendments and deviations to protocol will be submitted to LSTM’s REC for approval. The end of the study will be defined as the point at which all data collection, analysis, and reporting activities have been completed in accordance with the study protocol. Upon completion, the study will undergo formal close down procedures, including notifying the Sponsor, REC, and any other relevant regulatory bodies. This will involve submitting a final study report and confirming that all study activities have concluded. Archiving arrangements will follow institutional guidelines, in accordance with LSTM, LSHTM and BSMS’s respective policies, to ensure compliance with regulatory and ethical standards.

## Conclusion

This paper introduces the FLIGHT project and describes in detail the methods we will employ to achieve the first of four overarching project goals. After the study’s baseline assessment is completed (goal 1), we intend to develop and implement a suite of interventions including training programs, and other changes to policies and processes at partner HEIs. We hope the resulting insights and subsequent interventions will improve our respective institutional capability to accelerate the development of our global health-facing R&D assets. We will consolidate our methodology and (research) project outputs into an innovation and enterprise framework (IEF) as a model of best practice for global health (and impact-driven) research commercialisation in the UK and globally.

## Supporting information

S1 File FLIGHT project survey questionnaire. (PDF)

S2 File FLIGHT project interview guide. (PDF)

S3 File FLIGHT project participant information sheet. (PDF)

S4 File FLIGHT project consent form. (PDF)

S5 File FLIGHT project focus group interview guide. (PDF)
Acknowledgements
The authors would like to thank Aaron Argomandkhah for designing the figures included in this publication.
