# Supplementary material for: Developing an innovation and enterprise framework for translating UK-driven global health research into commercially viable interventions: the FLIGHT study protocol
Source: PLoS One. 2025 May 13;20(5):e0323168. doi: 10.1371/journal.pone.0323168 (PMC12074321; doi:10.1371/journal.pone.0323168)
Supplement: S1 File — (PDF) [file pone.0323168.s001.pdf]

FLIGHT LSTM Study Survey

**Please select the relevant type of Employment:**		
Academic/Research	Technical	Professional Services

**Do you consider yourself an Early Career Researcher?**	
Yes	No

**Please select your department:**			
Vector Biology	International Public Health	Clinical Sciences	Tropical Disease Biology

**Have you been involved with any of the following processes: (Select all that are applicable)**			
Protecting Intellectual Property (IP) through patents, copyright, etc.	Licensing Intellectual Property	Setting up a Spinout Company	None (of the above)

**Commercialisation Culture**<br>This refers to the understanding and support available for growing the commercial applications of research and the opportunities available to researchers.		**Confidence**				
**On the scale, can you indicate how confident you feel...**<br>1 - Not Confident. 5 - Very confident.		**1**	**2**	**3**	**4**	**5**
**1.**	Understanding the commercialisations pathways available to academics.					
**2.**	Identifying how your work can be commercialised.					

**Please select from the options below where you would like to see more information or training:**			
Finding out the commercialisations projects that are happening in my area of research.	The Support & Mentorship available from my institution for commercialisation.	How to build a roadmap for commercialising my research.	The potential impacts of commercialisation projects on me and my wider work.

For non-academic professional staff:

**Is this relevant to your role?**		**Yes**	**No (Skip)**	**Confidence**				
**On the scale, how confident would you feel talking to a researcher about...**<br>1 - Not Confident. 5 - Very confident.				**1**	**2**	**3**	**4**	**5**
**1.**	Understanding the broader commercialisations pathways available to academics.							
**2.**	Identifying how their work can be commercialised.							
**Are there any issues or barriers that you feel are common in this area, particularly in relation to your role?**								
**Are there any aspects around commercialisation culture, where you would like to receive more training?**<br>***Please outline beneficial training topics for You/Your Team & for Researchers:***								

**Intellectual Property**<br>This refers to any creations or knowledge arising from research that could be legally protected, via patenting & copyright, etc.		**Confidence**				
**On the scale, can you indicate how confident you feel…**<br>1 - Not Confident. 5 - Very confident.		**1**	**2**	**3**	**4**	**5**
**1.**	Determining what constitutes Intellectual Property - what things can & can't be protected.					
**2.**	Recognising potential Intellectual Property within your own work.					
**3.**	Knowing when and where you can disclose your IP (or related research).					
**4.**	Understanding the School's policy around Intellectual Property, including ownership.					
**5.**	Knowing who to approach in the school to talk about & support Intellectual Property.					

Please select from the options below where you would like to see more information or training:

The benefits of protecting my Intellectual Property.	Assessing whether an idea is worth protecting - evaluating its potential value to other parties.	Finding out whether an idea has already been protected.	Which stage of the innovation process to secure Intellectual Property.
How to determine whether elements of intellectual property will need to be protected when consulting.	Types of Intellectual Property		

For non-academic professional staff:

**Is this relevant to your role your role?**		**Yes**	**No (Skip)**	**Confidence**				
**On the scale, how confident would you feel talking to a researcher about...**<br>1 - Not Confident. 5 - Very confident.				**1**	**2**	**3**	**4**	**5**
**1.**	Determining what constitutes Intellectual Property - what things can & can't be protected.							
**2.**	Recognising potential Intellectual Property within their work.							
**3.**	Knowing when and where they can disclose their IP (or related research).							
**4.**	Understanding the School's policy around Intellectual Property, including ownership.							
**5.**	Knowing who to approach in the school to talk about & support Intellectual Property.							
**Are there any issues or barriers that you feel are common in this area, particularly in relation to your role?**								
**Are there any aspects around intellectual property, where you would like to receive more training?**<br>***Please outline beneficial training topics for You/Your Team & for Researchers:***								

**Funding for Research Commercialisation**<br>This refers to the funding that is available to support commercialisation or collaboration with industry.<br>Research commercialisation is the process by which new or improved technologies, products, processes and services that have arisen from research are brought to market.		**Confidence**				
**On the scale, can you indicate how confident you feel…**<br>1 - Not Confident. 5 - Very confident.		**1**	**2**	**3**	**4**	**5**
**1.**	Finding potential funding opportunities.					
**2.**	How to successfully write funding bids to support research commercialisation.					
**3.**	Accessing support for grant funding at my school.					
**4.**	Working with confidentiality agreements & non-disclosure agreements.					

Please select from the options below where you would like to see more information or training:

Evaluating the commercial potential of projects – highlighting return on investment.	Budget/Grant Management best practice.	How to adapt my work for commercial audiences.	

For non-academic professional staff:

Is this relevant to your role your role?		Yes	No (Skip)	Confidence				
**On the scale, how confident would you feel talking to a researcher about...**<br>1 - Not Confident. 5 - Very confident.				1	2	3	4	5
**1.**	Finding potential funding opportunities.							
**2.**	How to successfully write funding bids to support research commercialisation.							
**3.**	Accessing support for grant funding at my school.							
**4.**	Working with confidentiality agreements & non-disclosure agreements.							
**Are there any issues or barriers that you feel are common in this area, particularly in relation to your role?**								
**Are there any aspects around commercialisation funding, where you would like to receive more training?**<br>***Please outline beneficial training topics for You/Your Team & for Researchers:***								

**Licensing your Intellectual Property**<br>This is where an external organisation is given the right to use intellectual property.		**Confidence**				
**On the scale, can you indicate how confident you feel…**<br>1 - Not Confident. 5 - Very confident.		**1**	**2**	**3**	**4**	**5**
**1.**	Understanding the process of licensing Intellectual Property.					
**2.**	Understanding the benefits and drawbacks of licensing intellectual property					
**3.**	How to access Licensing support from the School & who to approach.					

Please select from the options below where you would like to see more information or training:

Effectively communicating the commercial potential of my Intellectual Property.	Different Types of Licencing agreements.	Where to find a licensing partner.	How to assess the resources, intent & viability of potential partners.

For non-academic professional staff:

Is this relevant to your role your role?		Yes	No (Skip)	Confidence				
**On the scale, how confident would you feel talking to a researcher about…**<br>1 - Not Confident. 5 - Very confident.				**1**	**2**	**3**	**4**	**5**
**1.**	Understanding the process of licencing Intellectual Property.							
**2.**	Understanding the benefits and drawbacks of licencing intellectual property							
**3.**	How to access Licensing support from the School & who to approach.							
**Are there any issues or barriers that you feel are common in this area, particularly in relation to your role?**								
**Are there any aspects around Licensing, where you would like to receive more training?**<br>***Please outline beneficial training topics for You/Your Team & for Researchers:***								

<table>
<tr><td colspan="2">Spinouts
This is where a researcher will start a company, based on their research, which is part owned by the University.</td><td colspan="5">Confidence</td></tr>
<tr><td colspan="2">On the scale, can you indicate how confident you feel…
1 - Not Confident. 5 - Very confident.</td><td>1</td><td>2</td><td>3</td><td>4</td><td>5</td></tr>
<tr><td>1.</td><td>Understanding the Spinout process, including the benefits and drawbacks.</td><td></td><td></td><td></td><td></td><td></td></tr>
<tr><td>2.</td><td>The impact of a spinout on your wider work, as well as your work-life balance.</td><td></td><td></td><td></td><td></td><td></td></tr>
<tr><td>3.</td><td>The School’s Spinout policy – how much of the company will be owned by the University.</td><td></td><td></td><td></td><td></td><td></td></tr>
</table>

**Please select from the options below where you would like to see more information or training:**			
The process of creating a spinout company from research.	Pitching a business idea	Setting up and running a business.	Where to find potential funding for a Spinout.

For non-academic professional staff:

**Is this relevant to your role your role?**		**Yes**	**No (Skip)**	**Confidence**				
**On the scale, how confident would you feel talking to a researcher about…**<br>1 - Not Confident. 5 - Very confident.				**1**	**2**	**3**	**4**	**5**
**1.**	Understanding the Spinout process, including the benefits and drawbacks.							
**2.**	The impact of a spinout on your wider work, as well as your work-life balance.							
**3.**	The School's Spinout policy – how much of the company will be owned by the University.							
**Are there any issues or barriers that you feel are common in this area, particularly in relation to your role?**								
**Are there any aspects around spinouts, where you would like to receive more training?**<br>***Please outline beneficial training topics for You/Your Team & for Researchers:***								

**Professional Skills**<br>This refers to the broader professional skills that might useful when working on commercialisation projects.		**Confidence**				
**On the scale, can you indicate how confident you feel…**<br>1 - Not Confident. 5 - Very confident.		**1**	**2**	**3**	**4**	**5**
**1.**	In your own ability or self-confidence, to take the first steps towards commercialising your research.					
**2.**	In your ability to self-manage and keep motivated on a potentially long-term commercialisation project.					

Please select from the options below where you would like to see more information or training:

Finding networking, public & commercial engagement opportunities.	Adapting communication style to non-academic audiences.	Building & maintaining relationships with partners.	Managing different stakeholders on a project.
Data Management, Control & Protection when working with an external partner.			

For non-academic professional staff:

Is this relevant to your role your role?		Yes	No (Skip)	Confidence				
**On the scale, how confident would you feel talking to a researcher about…**<br>1 - Not Confident. 5 - Very confident.				1	2	3	4	5
**1.**	In your own ability or self-confidence, to take the first steps towards commercialising your research.							
**2.**	In your ability to self-manage and keep motivated on a potentially long-term commercialisation project.							
**Are there any issues or barriers that you feel are common in this area, particularly in relation to your role?**								
**Are there any professional skills that you would like to see more training available?**<br>***Please outline beneficial training topics for You/Your Team & for Researchers:***								
