# Supplementary material for: Developing an innovation and enterprise framework for translating UK-driven global health research into commercially viable interventions: the FLIGHT study protocol
Source: PLoS One. 2025 May 13;20(5):e0323168. doi: 10.1371/journal.pone.0323168 (PMC12074321; doi:10.1371/journal.pone.0323168)
Supplement: S2 File — (PDF) [file pone.0323168.s002.pdf]

FLIGHT Baseline Assessment Interview Guide

Interview Introduction

"Thank you for joining us today. We really appreciate you taking the time to speak with us.

Our conversation is part of the FLIGHT project, which is dedicated to empowering the commercialisation of global health research at LSTM/LSHTM/BSMS.

We're eager to hear your experiences and thoughts on how we can better support these efforts. Your insights will play a key role in shaping new approaches and frameworks, not just for our school, but potentially for universities everywhere.

We hope this will be a relaxed and open conversation, and we're incredibly grateful for your willingness to share your perspectives with us."

For Interviewer: Rubric Scoring

Rubric scoring is a method that uses a predefined set of criteria to evaluate performance or achievement, assigning scores based on how well specific standards are met within each criterion.

Total Score Calculation: For the **24 questions**, the total score will range from **24 (minimum) to 120 (maximum)**.

The overall awareness or understanding score of a participant can be expressed using a classification category:

- 108–120: High Awareness (90–100%)
- 84–107: Mostly Aware (70–89%)
- 60–83: Moderate Awareness (50–69%)
- 36–59: Limited Awareness (25–49%)
- 24–35: Minimal Awareness (0–24%)

Understanding and Experience

1. **What is your understanding of Enterprise and Innovation? and what has informed your understanding?**

**Score**	**Description**	Score
**5**	The participant has a thorough understanding of scientific commercialisation, informed by extensive professional experience, training, or education. They consider themselves highly knowledgeable and experienced in the field.	☐
**4**	The participant has a solid understanding of enterprise and innovation, informed by some experience or training, but considers themselves more knowledgeable than experienced.	☐
**3**	The participant has a basic understanding of enterprise and innovation, informed by limited exposure or informal sources. They do not consider themselves highly knowledgeable or experienced.	☐
**2**	The participant has heard of enterprise and innovation but lacks a clear understanding and has minimal exposure or experience in this area.	☐
**1**	The participant has no understanding of enterprise and innovation and no experience in this area.	☐

2. **Based on your understanding of enterprise activities, what is your understanding of scientific commercialisation? What has informed your understanding?**

**Score**	**Description**	Score
**5**	The participant has a thorough understanding of scientific commercialisation (licensing/spin out/ business development etc), informed by extensive professional experience, training, or education. They consider themselves highly knowledgeable and experienced in the field.	☐
**4**	The participant has a solid understanding of scientific commercialisation (licensing/spin out/ business development etc), informed by some experience or training, but considers themselves more knowledgeable than experienced.	☐
**3**	The participant has a basic understanding of scientific commercialisation (licensing/spin out/ business development etc), informed by limited exposure or informal sources. They do not consider themselves highly knowledgeable or experienced.	☐
**2**	The participant has heard of scientific commercialisation (licensing/spin out/ business development etc) but lacks a clear understanding and has minimal exposure or experience in this area.	☐
**1**	The participant has no understanding of scientific commercialisation (licensing/spin out/ business development etc) and no experience in this area.	☐

3. **Do you know how scientific commercialisation-related activities are currently supported in your institution?**

Score	Description	Score
**5**	The participant has a detailed understanding of how scientific commercialisation are supported at their institution and can describe specific resources, programs, and strategies in place.	☐
**4**	The participant has a good understanding of the support available for scientific commercialisation at their institution but is less familiar with the full range of resources or programs.	☐
**3**	The participant is aware that there is support for scientific commercialisation but lacks specific knowledge of how it operates within their institution.	☐
**2**	The participant is vaguely aware that some support for scientific commercialisation exists but does not know any details about it.	☐
**1**	The participant is unaware that their institution provides any support for scientific commercialisation.	☐

4. **In your experience, what are the main strengths and weaknesses of the current support for enterprise and innovation at your institution?**

Score	Description	Score
**5**	The participant can clearly articulate the strengths, weaknesses, and gaps in the support system at their institution, based on in-depth experience with these resources.	☐
**4**	The participant has a good understanding of the strengths and weaknesses of the support system but may have less clarity on specific gaps or areas for improvement.	☐
**3**	The participant has a general sense of the support system's strengths and weaknesses but struggles to identify specific gaps.	☐
**2**	The participant has limited awareness of the strengths and weaknesses of the support system and cannot identify clear gaps.	☐

**1**	The participant is unable to identify any strengths, weaknesses, or gaps in the support provided, due to lack of awareness or engagement with the system.	☐

Reward, Recognition and Incentives

5. **In what ways are innovation successes currently rewarded, recognised, or incentivised within the school?**

**Score**	**Description**	Score
**5**	The participant is aware of multiple formal mechanisms for rewarding and recognising innovation successes, including monetary, public acknowledgment, or career advancement.	☐
**4**	The participant is aware of some formal recognition or incentives but believes more could be done to improve the system.	☐
**3**	The participant has limited awareness of any formal mechanisms for recognition or incentives within the school.	☐
**2**	The participant is vaguely aware of any rewards or recognition but cannot name specific programmes or processes.	☐
**1**	The participant believes there is no formal mechanism for rewarding or recognising innovation successes.	☐

6. **At present, do you feel research translation activities are a priority for the school to improve health outcomes in disadvantaged populations globally?**

**Score**	**Description**	Score
**5**	The participant believes that innovation/commercialisation outputs are highly valued and clearly prioritised.	☐
**4**	The participant feels these outputs are somewhat valued but not given the same level of importance as traditional research outputs.	☐
**3**	The participant feels innovation outputs are moderately valued but are secondary to other academic activities.	☐
**2**	The participant believes these outputs are not strongly valued or prioritised within the school.	☐

**1**	The participant believes that innovation outputs are undervalued and do not align with the school's priorities.	☐

7. **Enterprise and Innovation activities, such as research translation, commercialisation, and contracts are typically not a focus within academic career tracks.**

   Should greater emphasis be placed on these activities for career progression within academia?

   If non-academic professional staff, ask instead –
   Enterprise and Innovation activities, such as research translation, commercialisation, and contracts are often concentrated within certain departments.

   Should these activities be integrated more widely across all areas of the institution to enhance visibility and cross-departmental engagement?

**Score**	**Description**	Score
**5**	The participant believes the lack of focus on enterprise and innovation is a significant issue and should be mandated for career progression.	☐
**4**	The participant sees the absence of enterprise and innovation in career progression as an issue but is unsure about making it a mandate.	☐
**3**	The participant recognises that enterprise and innovation are secondary considerations but does not see this as a major issue.	☐
**2**	The participant does not feel strongly about this issue but sees value in discussing potential changes.	☐
**1**	The participant does not believe that enterprise and innovation should be a focus of an academic career track.	☐

8. **Would an award scheme encourage greater commercialisation of research within the school? If yes, why and how might this be structured/implemented?**

**Score**	**Description**	Score
**5**	The participant strongly supports the idea of an award scheme and has clear ideas for how it should be structured and implemented.	☐
**4**	The participant supports the idea but is unsure how best to structure or implement such a scheme.	☐
**3**	The participant thinks an award scheme could be useful but does not have strong opinions on its necessity or implementation.	☐
**2**	The participant is unsure whether an award scheme would have an impact.	☐
**1**	The participant believes an award scheme would not encourage greater commercialisation of research.	☐

9. **Would you support more commercialisation learning opportunities, such as workshops, events, and webinars, being promoted across the school to enhance staff access to commercialisation support?**

**Score**	**Description**	Score
**5**	Participant is highly enthusiastic about expanding commercialisation support and is eager to actively engage in workshops, events, and webinars.	☐
**4**	Participant is enthusiastic about additional commercialisation learning opportunities and is likely to participate when available.	☐
**3**	Participant is moderately interested and willing to engage if the opportunities align with their interests or availability.	☐
**2**	Participant is not particularly enthusiastic and unsure of the relevance; unlikely to engage unless specifically needed.	☐
**1**	Participant is not interested in expanded commercialisation support and has no intention of engaging in related activities.	☐

10. What would you like to happen so that as a PI your academic outputs such as publishing are not hindered by commercialisation activities?

If non-academic professional staff, ask instead –
What would you like to see happen to support commercialisation without hindering your professional outputs?

**Score**	**Description**	Score
**5**	The participant has clear suggestions for how to balance academic/professional outputs with commercialisation, believing it is crucial for their success.	☐
**4**	The participant has some ideas on how to prevent commercialisation activities from hindering academic/professional outputs but is unsure of their feasibility.	☐
**3**	The participant feels there is a moderate impact of commercialisation activities on academic/professional outputs but does not have strong ideas for change.	☐
**2**	The participant does not believe commercialisation activities significantly hinder academic/professional outputs.	☐
**1**	The participant believes that academic/professional outputs are not affected by commercialisation activities.	☐

11. **Are there specific incentives that would motivate you to engage with the commercialisation process?**

Score	Description	Score
5	The participant can identify specific incentives that would strongly motivate them to engage with commercialisation activities.	☐
4	The participant can identify some incentives that would encourage them but is uncertain about their effectiveness.	☐
3	The participant sees some value in incentives but is not particularly motivated by them.	☐
2	The participant feels that incentives would not have a significant impact on their decision to engage.	☐
1	The participant believes that incentives would not influence their engagement with the commercialisation process.	☐

12. **How likely would you be to engage with an innovation hub offering networking events, support & activities, drop-in sessions for commercialisation?**

Score	Description	Score
5	The participant is highly likely to engage with an innovation hub and sees it as a valuable resource.	☐
4	The participant is likely to engage but may need more information on how the hub operates.	☐
3	The participant sees some value in engaging but is not fully committed.	☐
2	The participant is unlikely to engage but is open to learning more.	☐
1	The participant is highly unlikely to engage with an innovation hub.	☐

Policy and Processes

13. Would you know how to investigate the potential to protect intellectual property that has been generated?

Score	Description	Score
5	The participant has a clear understanding of the process, knows where to access IP resources, and has previously engaged in IP or patent filing at the institution.	☐
4	The participant is aware of how to investigate IP potential and knows where resources are but hasn't personally engaged in the process.	☐
3	The participant knows that IP resources exist but is unsure where to find them or how to engage with the process.	☐
2	The participant is vaguely aware of the need for IP but has little to no understanding of how to investigate IP potential.	☐
1	The participant is completely unaware of the process or that IP are relevant to their work.	☐

14. **Do you have access to resources and information on how to collaborate with industry partners?**

Score	Description	Score
5	The participant has full access to industry collaboration guidance and has used it multiple times. They know exactly who to approach and what procedures to follow.	☐
4	The participant has access to guidance and has occasionally used it but may not be fully familiar with every aspect.	☐
3	The participant knows guidance exists but has not used it, or only has limited access.	☐
2	The participant is unsure if any guidance on industry collaboration is available.	☐
1	The participant is unaware of any guidance or resources for working with industry.	☐

15. **Do you consider protecting intellectual property throughout the course of research projects? How do you do this?**

Score	Description	Score
5	The participant regularly assesses the IP potential of their research and actively engages in processes to protect it when applicable.	☐
4	The participant often considers whether their research has IP potential but may not always act to protect it.	☐
3	The participant sometimes assumes IP potential but does not consistently evaluate or protect it.	☐
2	The participant rarely considers whether their research has IP to protect.	☐
1	The participant never considers IP protection in their research.	☐

16. **Do you know how we ensure global access to our translational projects?**

Score	Description	Score
5	The participant is very familiar with the institution's global access policies and has contributed to ensuring global access in their work.	☐
4	The participant knows the institution has a global access policy and understands the general process for ensuring global access.	☐
3	The participant has heard of global access initiatives but is unclear on the details or how to ensure it themselves.	☐
2	The participant is vaguely aware of global access but does not know how it's managed at the institution.	☐
1	The participant is unaware of global access policies, or any processes related to it.	☐

17. When would you consider a Non-Disclosure Agreement (NDA) is needed in discussions?

Score	Description	Score
5	The participant is fully aware of when NDAs are required, regularly engages legal teams, and actively uses NDAs in relevant discussions.	☐
4	The participant understands the general situations where an NDA is needed but may not always engage with legal teams early on.	☐
3	The participant has a basic understanding of NDAs but may not actively use them unless prompted.	☐
2	The participant has little knowledge of when NDAs are needed and rarely considers them.	☐
1	The participant is unaware of what an NDA is or why it would be required.	☐

18. How comfortable do you feel seeking training on topics like commercialisation, intellectual property, patents, and agreements? If you were interested, how would you likely engage with this training, if offered?

Score	Description	Score
5	The participant actively seeks out training, knows who to approach, and regularly engages in institutional training programs.	☐
4	The participant is aware of training opportunities and who to approach but may not engage regularly.	☐
3	The participant knows training is available but does not actively seek it or may not know who to contact.	☐
2	The participant has limited awareness of training resources and rarely seeks out training.	☐
1	The participant is unaware of any training opportunities and does not know who to approach.	☐

Sustainability of commercialisation initiatives

19. **How do you believe we should grow innovation initiatives at LSTM?**

**Score**	**Description**	Score
**5**	The participant has a detailed understanding of growth and can suggest specific, actionable ways to sustain innovation at LSTM.	☐
**4**	The participant has some ideas for growth and sustainability but is unsure of how to implement them.	☐
**3**	The participant has a general sense of growth but lacks specific, detailed ideas.	☐
**2**	The participant is aware of the need for growth but has few ideas for sustaining innovation.	☐
**1**	The participant has no clear ideas on how to grow or sustain innovation initiatives.	☐

20. **What specific goals or milestones would you like to see achieved in the innovation process at LSHTM over the next 5–10 years?**

**Score**	**Description**	Score
**5**	The participant outlines clear, specific goals and milestones for innovation at LSTM, with a strong vision for the next 5–10 years.	☐
**4**	The participant suggests some specific goals but lacks clarity or depth on certain milestones.	☐
**3**	The participant identifies broad goals but lacks detailed milestones or a clear long-term vision.	☐
**2**	The participant expresses vague ideas about goals and milestones with little specificity.	☐
**1**	The participant offers no goals or milestones and has limited vision for innovation in the future.	☐

21. **In what ways do you think the revenues from commercial ventures should be best reinvested to ensure the ongoing success of innovation activities at LSTHM?**

**Score**	**Description**	Score
**5**	The participant presents specific and well-thought-out reinvestment strategies that directly support the sustainability of innovation activities.	☐
**4**	The participant suggests relevant reinvestment areas but lacks some details on how this would support long-term sustainability.	☐
**3**	The participant provides some general ideas on reinvestment but lacks clear links to sustainability of innovation activities.	☐
**2**	The participant is unsure or vague about reinvestment strategies and how they would contribute to sustainability.	☐
**1**	The participant has minimal or no suggestions for reinvesting revenues to support innovation sustainability.	☐

Knowledge of Enterprise and Innovation

22. **Would you welcome communication and updates from the Enterprise and Innovation team around opportunities to collaborate? If so, what channels are you most likely to engage with?**

**Score**	**Description**	Score
**5**	The participant is very enthusiastic about receiving communications and suggests multiple channels they regularly engage with.	☐
**4**	The participant is open to communications and lists a couple of preferred channels but is less specific.	☐
**3**	The participant is somewhat interested but does not express a strong preference for communication channels.	☐
**2**	The participant is not particularly engaged and shows little interest in regular communication.	☐
**1**	The participant is not interested in communications or collaboration updates.	☐

23. **Are you aware that wider funding opportunities could be developed through engaging with the innovation and enterprise team, with the potential to broaden your research scope?**

**Score**	**Description**	Score
**5**	The participant is fully aware of wider funding opportunities and actively seeks to engage with the enterprise and innovation agenda.	☐
**4**	The participant is aware of funding opportunities but has not yet engaged with the enterprise and innovation agenda.	☐
**3**	The participant has some awareness of funding opportunities but is unsure how to engage with the agenda.	☐
**2**	The participant has limited awareness of funding opportunities related to the enterprise and innovation agenda.	☐
**1**	The participant is completely unaware of funding opportunities linked to the enterprise and innovation agenda.	☐

24. **How well do you understand the specific ways the Innovation Team can assist with developing research into real-world applications? Are there any types of support or learning you feel would be valuable in advancing your work?**

**Score**	**Description**	Score
**5**	The participant is very familiar with the E&I's support for research commercialisation and is actively engaged.	☐
**4**	The participant is aware of the E&I's support but has not engaged much and would like to learn more.	☐
**3**	The participant has some familiarity with the E&I's work but lacks details and would benefit from further information.	☐
**2**	The participant is vaguely aware of the E&I's role and its support for commercialisation.	☐
**1**	The participant is not familiar with the E&I and its support for research commercialisation.	☐

Closing Question

Thank you for your time and valuable insights. Before we close this interview, do you have any additional comments or thoughts on commercialisation that you'd like to share?

Overall Awareness Score (/120)
