# Supplementary material for: Developing an innovation and enterprise framework for translating UK-driven global health research into commercially viable interventions: the FLIGHT study protocol
Source: PLoS One. 2025 May 13;20(5):e0323168. doi: 10.1371/journal.pone.0323168 (PMC12074321; doi:10.1371/journal.pone.0323168)
Supplement: S3 File — (PDF) [file pone.0323168.s003.pdf]

Participant Information Sheet – Interviews

**Study Title: Framework for Leveraging Innovation in Global Health Technologies (FLIGHT) Interviews**	
**Principal Investigator:**<br><br>**Dr Becky Jones-Philips**<br>Head of Enterprise and Innovation<br>Becky.Jones-Phillips@lstmed.ac.uk<br>+44(0)151 705 3100	**Study Sites:**<br><br>**Liverpool School of Tropical Medicine (LSTM)**<br><br>**London School of Hygiene and Tropical Medicine (LSHTM)**<br><br>**Brighton and Sussex Medical School (BSMS)**

Introduction

You are being invited to participate in a research study by talking to us in a semi-structured interview, exploring your familiarity with the research commercialisation processes at LSTM. Before you decide whether to participate, it is important for you to understand why the research is being done and what it will involve. Please take time to read the following information carefully, feel free to contact the Information Governance team if you would like more information or if there is anything that you do not understand. We would like to stress that you do not have to accept this invitation and should only agree to take part if you want to.

What is the purpose of the Interview?

The FLIGHT programme has been set up to explore and expand how the commercialisation of global health research might be better supported across the school, and to create a best practice framework for other universities to follow.
A large focus of this programme is allocated to improving the support offered for commercialisation projects across the school.

The following interview aims to gather insights into your experience around commercialisation processes, and how you feel the school might better support these. This will be used to inform new processes that can be introduced to enhance understanding and confidence in carrying out commercialisation projects.

Why have I been chosen to take part?
This study will be looking to gain insights from a wide range of staff at LSTM. This is to engage a wide range of perspectives from people with diverse levels of experience & familiarity with research commercialisation.

Do I have to take part?
Participation is completely voluntary, and participants are free to withdraw at any time before or after the interview.

If you choose to participate in the study, please notify the study team within three weeks of receiving your enrolment information.

What will happen if I take part?
The interview will follow a semi-structured format, where we will invite you to talk about your experiences relating to different processes of research commercialisation. Your responses will be used to inform us of any areas where support could be introduced or strengthened for LSTM staff interested in commercialising their research.

The anonymised information from this will be used within publications from the FLIGHT programme.

How will my data be used?
1. How will my data be collected?

The interview will last for 1 hour and held in-person, and Microsoft Teams will be used if the participant requires the meeting to be online.

For both in-person and online interviews, a recording will be taken via Microsoft Teams 'Record Meeting' function. Some interview responses will also be captured in written form on a data collection sheet by a research team member/note taker.

2. How will my data be stored?

Interview recordings and transcripts will be stored securely on an LSTM password protected computer's OneDrive and in the FLIGHT team's SharePoint folder and will only be accessible to the research team.

3. How long will my data be stored for?

The recordings will be deleted immediately after transcription and other study materials (consent forms, transcripts, etc) will be stored securely for up to 5 years before deletion

in accordance with LSTM's Research Governance & Ethics Office data storage recommendations/procedures.

4. What measures are in place to protect the security and confidentiality of my data?

The transcribed data will be cleaned, removing any information that might potentially identify the participant, keeping only information specific to the purpose of the FLIGHT programme. The participant will be provided a copy of the recording and transcript, to provide the respondent an opportunity to confirm that their interview responses have been correctly and confidentially captured.

5. Will my data be anonymised?

All data will be anonymised, with no personal data being taken. Only relevant specific information will be stored as data, with references to specific projects or events being removed.

6. How will my data be used?

The data from responses will be analysed to show trends across *LSTM in relation to the level of support offered for research commercialisation activities. This will inform the development of processes and support for LSTM staff members.*

7. Who will have access to my data?

The FLIGHT Team at LSTM (Dr Becky Jones-Philips, Dr Chris Peters, Dr Ezekiel Boro, Charles McLoughlin, Tom Vaughan, Dr Carolina Velasco).

8. Will my data be archived for use in other research projects in the future?

There are no plans to make this data available for reuse, however, there is intention that the data from this survey will be used within publications from the FLIGHT programme.

Are there any risks in taking part?
There are limited risks to the participants in using this research method, though these will be mitigated with the anonymisation of transcribed data and subsequent deletion of recordings. They can withdraw from the study at any time, and they do not need to provide a reason for doing so. The transcribed data from the interview will be completely anonymous and will not include any personal data or special category data. The interview questions relate to experiences around research commercialisation processes at LSTM: The questions do not require the disclosure of sensitive information,

however, should any such information be volunteered, this will be removed from the transcript data. Due to the design & focus of the study, there is potential for participants to volunteer information that is critical of LSTM's procedures: These will be moderated to ensure that the feedback is constructive and that participants cannot be identified by their experiences. All responses will be anonymous, with data removed that might be identifiable to line-managers, department heads or other senior members of staff.

Are there any benefits in taking part?

This research will help LSTM understand the gaps in the support available for research commercialisation activities. This will be used to identify possible future support that might be introduced across the school.

What will happen to the results of the interviews?

As well as being used to highlight potential areas in the school where support for research commercialisation can be introduced, the results will be used in published articles and reports related to the wider outcomes of the FLIGHT programme.

Participants' consent will be sought for any future contact regarding separate, ethically approved studies, as outlined in the following consent statement:

"I consent to being contacted in the future with an invitation to participate in a separate, ethically approved research study."

This ensures that participants will only be approached for further involvement if they have explicitly opted in for future research opportunities.

Findings from this study will be shared with the funder (Research England) and each institution's administration through internal reports. Results will also be disseminated at local (UK HEIs research & funders community) and international levels (via scientific conferences and academic journals). All dissemination of data will follow the guidelines of the International Committee of Medical Journal Editors (ICMJE) and authorship will include all individuals involved in the study and meeting the ICMJE criteria for authorship. At the conclusion of the study, a summary report of the results will be shared directly with participants through email or other agreed-upon communication methods. This ensures that participants remain fully informed of the study's outcomes, reinforcing transparency and recognising their valuable contribution.

What will happen if I want to stop taking part?

Participants can withdraw from the study at any time before the data has been transcribed, within one week of the interview. As the interview guide is anonymised from

the start, there is no way in which the researcher can identify who has submitted what responses.

What if I am unhappy or if there is a problem?
If you are unhappy, or if there is a problem, please feel free to let us know by contacting the FLIGHT Study PI & Safeguarding Lead: **Becky Jones-Philips** or any other member of the FLIGHT team and we will try to help. If you remain unhappy or have a complaint which you feel you cannot come to us with then you should contact the Research Ethics Team at lstmrec@lstmed.ac.uk. When contacting the Research Ethics Team, please provide details of the name or description of the study (so that it can be identified), the researcher involved and the details of the complaint you wish to make.

Who can I contact if I have further questions?

Charles McLoughlin – Innovation Capacity Strengthening Manager:
charles.mcloughlin@lstmed.ac.uk

Tom Vaughan – Knowledge Exchange Manager:
tom.vaughan@lstmed.ac.uk

Dr Jenny Hill – Chair, Research Ethics Committee
jenny.hill@lstmed.ac.uk OR lstmrec@lstmed.ac.uk
