# Supplementary material for: Developing an innovation and enterprise framework for translating UK-driven global health research into commercially viable interventions: the FLIGHT study protocol
Source: PLoS One. 2025 May 13;20(5):e0323168. doi: 10.1371/journal.pone.0323168 (PMC12074321; doi:10.1371/journal.pone.0323168)
Supplement: S4 File — (PDF) [file pone.0323168.s004.pdf]

Consent Form

CONFIDENTIAL

**Study Title: Framework for Leveraging Innovation in Global Health Technologies (FLIGHT) Interviews**	
**Principal Investigator: Dr Becky Jones-Philips**	**Study Site:**<br><br>**Liverpool School of Tropical Medicine, U.K.**

**If you agree with each statement, please INITIAL the box provided**	
1. I confirm I have read and understood the information sheet dated September 2024 (Version 2) for the above study. I have had the opportunity to consider the information, ask questions and have had these answered satisfactorily.	
2. I understand that participation in this study is voluntary and I am free to withdraw consent at any time, without giving a reason, without any penalties.	
3. I agree to participating in a structured interview, discussing my professional experiences and opinions, as part of this research study.	
4. I understand that data collected during the study may be looked at by individuals from or delegated by LSTM and from regulatory authorities. I give permission for these individuals to have access to my records.	
5. I understand that my personal data will be handled in accordance with UK Law.	
6. My data will remain in the UK for analysis.	
7. I consent to being contacted in the future with an invitation to participate in a separate, ethically approved research study	
8. I understand that once my data has been fully anonymised, it cannot be deleted.	
9. I understand that my personal data, including consent forms, will be securely retained for 5 years in accordance with LSTM policy	
10. I agree to take part in this study.	

Name of participant	Date	Signature
Name of person taking consent	Date	Signature

(When complete: 1 copy for participant; 1 copy (original) for research)
