# Supplementary material for: Developing an innovation and enterprise framework for translating UK-driven global health research into commercially viable interventions: the FLIGHT study protocol
Source: PLoS One. 2025 May 13;20(5):e0323168. doi: 10.1371/journal.pone.0323168 (PMC12074321; doi:10.1371/journal.pone.0323168)
Supplement: S5 File — (PDF) Acknowledgements The authors would like to thank Aaron Argomandkhah for designing the figures included in this publication. [file pone.0323168.s005.pdf]

FLIGHT Baseline Assessment Focus Group Discussion Guide

Focus Group Discussion Introduction

"Thank you for joining us today. We really appreciate you taking the time to speak with us.

Our conversation is part of the FLIGHT project, which is dedicated to empowering the commercialisation of global health research at LSTM/LSHTM/BSMS.

We're eager to hear your experiences and thoughts on how we can better support these efforts. Your insights will play a key role in shaping new approaches and frameworks, not just for our school, but potentially for universities everywhere.

We hope this will be a relaxed and open conversation, and we're incredibly grateful for your willingness to share your perspectives with us."

Understanding and Experience

1. **What is your understanding of Enterprise and Innovation? and what has informed your understanding?**
2. **Based on your understanding of enterprise activities, what is your understanding of scientific commercialisation? What has informed your understanding?**
3. **Do you know how scientific commercialisation-related activities are currently supported in your institution?**
4. **In your experience, what are the main strengths and weaknesses of the current support for enterprise and innovation at your institution?**

Reward, Recognition and Incentives

5. **In what ways are innovation successes currently rewarded, recognised, or incentivised within the school?**
6. **At present, do you feel research translation activities are a priority for the school to improve health outcomes in disadvantaged populations globally?**
7. **Enterprise and Innovation activities, such as research translation, commercialisation, and contracts are typically not a focus within academic career tracks.**

Should greater emphasis be placed on these activities for career progression within academia?

8. Would an award scheme encourage greater commercialisation of research within the school? If yes, why and how might this be structured/implemented?
9. Would you support more commercialisation learning opportunities, such as workshops, events, and webinars, being promoted across the school to enhance staff access to commercialisation support?
10. What would you like to happen so that as an Early Career Researcher your academic outputs such as publishing are not hindered by commercialisation activities?
11. Are there specific incentives that would motivate you to engage with the commercialisation process?
12. How likely would you be to engage with an innovation hub offering networking events, support & activities, drop-in sessions for commercialisation?

Policy and Processes

13. Would you know how to investigate the potential to protect intellectual property that has been generated?
14. Do you have access to resources and information on how to collaborate with industry partners?
15. Do you consider protecting intellectual property throughout the course of research projects? How do you do this?
16. Do you know how we ensure global access to our translational projects?
17. When would you consider a Non-Disclosure Agreement (NDA) is needed in discussions?
18. How comfortable do you feel seeking training on topics like commercialisation, intellectual property, patents, and agreements? If you were interested, how would you likely engage with this training, if offered?

Sustainability of commercialisation initiatives

19. How do you believe we should grow innovation initiatives at the school?
20. What specific goals or milestones would you like to see achieved in the innovation process at the school over the next 5–10 years?

21. In what ways do you think the revenues from commercial ventures should be best reinvested to ensure the ongoing success of innovation activities at the school?

Knowledge of Enterprise and Innovation

22. Would you welcome communication and updates from the Enterprise and Innovation team around opportunities to collaborate? If so, what channels are you most likely to engage with?
23. Are you aware that wider funding opportunities could be developed through engaging with the innovation and enterprise team, with the potential to broaden your research scope?
24. How well do you understand the specific ways the Innovation Team can assist with developing research into real-world applications? Are there any types of support or learning you feel would be valuable in advancing your work?

Closing Question

Thank you for your time and valuable insights. Before we close this interview, do you have any additional comments or thoughts on commercialisation that you'd like to share?
